# A Practical Guide to Grade Adjustment or Curving for Pharmacy and Other Professional Health Programs

**DOI:** 10.3390/pharmacy13010004

**Published:** 2025-01-10

**Authors:** Reza Mehvar

**Affiliations:** Department of Biomedical and Pharmaceutical Sciences, School of Pharmacy, Chapman University, Irvine, CA 92618, USA; mehvar@chapman.edu

**Keywords:** grade adjustment, grade curve, curving grades, grade moderation, statistical moderation of grades

## Abstract

The peer-reviewed literature on the adjustment or curving of assessments in health profession programs is almost non-existent. This communication aims to present potential methods of grade adjustment for individual questions or entire assessments. Simulated data for a 25-item assessment were used as an example to analyze the effects of different methods of grade adjustment on students’ scores. Grade adjustments were made by adjusting the points for individual questions or the scores for the entire assessment. Adjustment for the individual questions was carried out by dropping the question, adding points to those who missed the question, or adding a bonus point to all students. Grade adjustment methods for the entire assessment included adjusting the mean or mean plus distribution (i.e., standard deviation) of the assessment score. Different methods of grade adjustments or curving for individual questions or the entire assessment resulted in drastically different outcomes for individual students’ scores. The justifications for selecting the appropriate method for adjustment of the individual scores are presented based on item analysis statistics. Curving or adjusting the score for the entire exam may be justified when there is a need for consistency in grade distribution among the assessments across the years or different sections of the course. Although methods for adjustment of grades are relatively easy to implement, instructors should have reasonable educational justification for deciding whether to adjust grades or which method to use.

## 1. Introduction

Although grades are currently an integral part of higher education as a measure of the scholastic achievement of students, they were not widely used until the 1940s [1]. Despite their widespread use in recent decades, grades and their relevance to student learning are a topic of significant debate and controversy [1,2,3,4,5]. Different instructors and institutions purportedly use grades for different purposes, such as feedback on performance, a tool for comparing students, an objective evaluation of student knowledge, or a motivator of student effort [1]. However, the validity of these uses has been seriously questioned by educators [1]. Notwithstanding their shortcomings, grades are currently a major determinant of admission, progression, and award of scholarships in most higher-education institutions, including health profession programs.

In professional medicine and pharmacy programs, recent movements have focused on deemphasizing grades by moving from numerical or letter grades to pass/fail grading [6,7] or competency-based education [8,9,10]. Whereas the pass/fail system is the dominant grading system used in Doctor of Medicine programs at US medical schools [7], the practice is currently adopted by only a handful of pharmacy schools in the US [6]. Additionally, competency-based education, which is an educational model that focuses on the outcome, has only recently been explored as a paradigm shift in health professions education, including pharmacy [8,9]. Therefore, currently, most Doctor of Pharmacy and graduate programs in biomedical and pharmaceutical sciences in the US and perhaps worldwide use summative assessments that result in assigning numerical scores or letter grades for each student.

Ideally, an assessment is developed based on a blueprint [11] with validated questions. Such ideal assessments do not necessarily need any grade adjustments or curving after scoring. However, in practice, instructors may use new unvalidated questions, which may require adjustment of grades based on rescoring individual questions or adjustment of the score for the entire exam. For individual questions, most software programs used for grading electronic or multiple-choice paper exams provide statistical data, such as measures of difficulty (item difficulty) and discrimination among test takers (e.g., point biserial) for each question [12,13,14]. The item difficulty ranges from 0, when no student answers the question correctly, to 1, when 100% of students answer the question correctly. The point biserial, on the other hand, is the correlation between the student response to the question (item score) and the student score on the entire assessment and is a measure of how well the item differentiates between the high and low performers in the test. The point biserial ranges from −1 to +1, with +1 indicating a perfect positive correlation, 0 indicating no correlation, and −1 indicating a negative correlation (poor performers in the test answering the question correctly). Generally, a negative point biserial suggests that the item is structurally flawed. The item difficulty and point biserial may be used to evaluate the validity of the individual questions and the need to adjust the grades by rescoring the individual questions [12,13,14]. Additionally, there may be situations that require grade adjustment or curving for the entire exam. These situations include adjusting the grades for different sections of the same course when different exams with varying degrees of difficulty are administered or when there is a desire to have consistency in grade distribution across the years or assessments.

A review of the literature for grade adjustment or curving indicates that most of the available literature focuses on “grading on the curve” [3,4,15,16,17,18,19], which is generally limited to undergraduate education. These publications specifically discuss how to distribute grades on a bell-shaped (normal distribution) curve for the specific purpose of limiting the number of grades in each category, such as assigning a limited number of A grades, even if most students performed at a very high level. This type of “grading on the curve” does not seem to be common or appropriate for higher-level graduate courses or professional programs, where grades are supposed to reflect student learning, with no a priori limitation on the number of students who may earn high grades.

In addition to “grading on the curve”, the subject of grade adjustment or “moderation” has been primarily addressed in the literature in the context of adjusting grades across different high schools for high school certificates [20,21,22,23] or for the prediction of academic performance or admission to undergraduate or graduate programs [24,25]. However, surprisingly, no peer-reviewed publications related to grade adjustment or grade curves in health profession programs could be identified. Therefore, the purpose of this communication is to discuss different methods to adjust exam grades based on adjusting or curving grades for individual questions and/or the entire exam without “grading on the curve” that limits the number of high scores in each assessment.

## 2. Materials and Methods

### 2.1. Data

**Figure 1:**
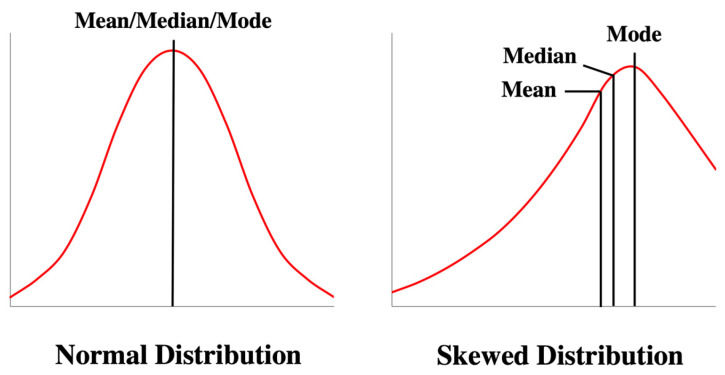
Distribution of grades for normal distribution (**left**) and left-skewed distribution (mean is to the left of median).

**Figure 2:**
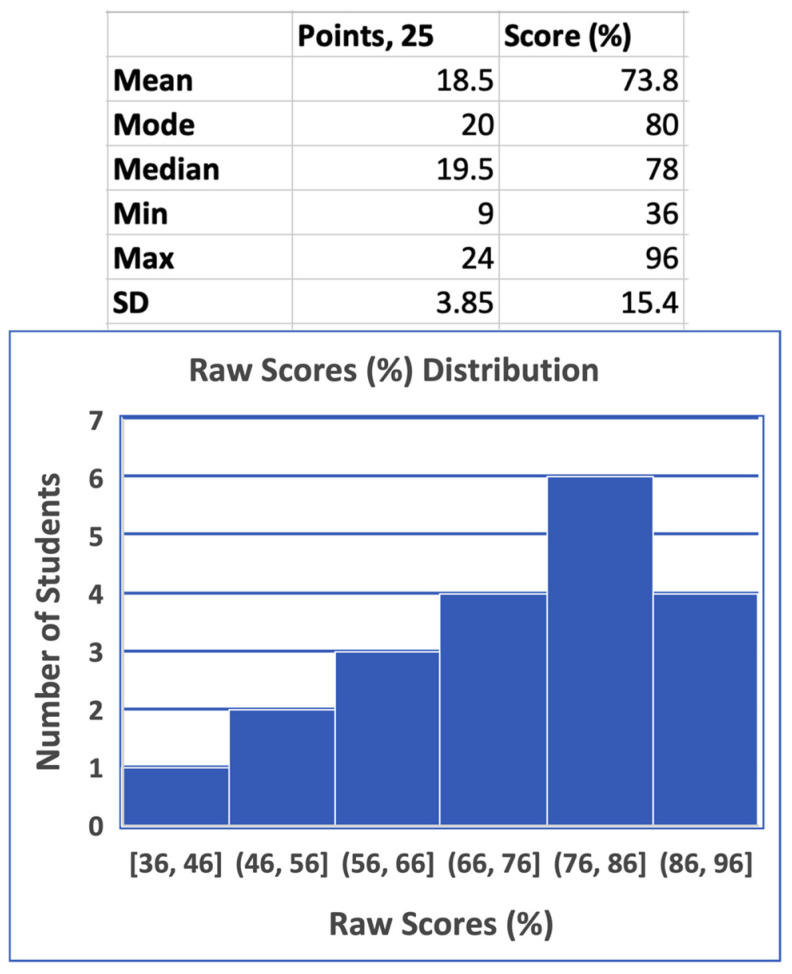
Descriptive statistics, including the distribution frequency of scores, for 20 students in a 25-item assessment with equal weight for all questions.

In most high-level undergraduate courses or graduate or professional programs, like pharmacy, the distribution of grades is skewed towards higher frequency for the higher grades (Figure 1) [3,26]. Therefore, grades were simulated for 20 students in a 25-item exam to mimic such skewed distribution (Figure 2), as seen with actual data for different courses at the author’s institution. All the items were assumed to have equal weights, with a total exam point of 25 or 100%. As demonstrated in Figure 2, grades range from the minimum (min) of 9 points (36%) to a maximum (max) of 24 points (96%), with an average (mean) of 73.8% and a standard deviation (SD) of 15.4%. As explained below, this set of raw grades was used to analyze different grade adjustment schemes for individual questions or the entire exam.

### 2.2. Grade Adjustment for Individual Questions

The raw data were used to adjust the grades in a hypothetical case when one of the questions was problematic, such as a question that was poorly structured or incorrect, had additional correct answers, or had a higher-than-desired degree of difficulty. Three different approaches were considered to resolve the problem, and the effects of each approach on the individual students’ scores in the exam were analyzed. The three different methods were: (1) dropping the question entirely, (2) considering the answers of all students to the question as correct, and (3) adding a point to all students, including those who had already received credit for the question.

### 2.3. Grade Adjustment for the Entire Exam

Grade adjustment for the entire exam could be achieved by adjusting mean only or adjusting both mean and the distribution (SD) of grades using the following equation [20]:

(1) $$Adjusted\;Score=Adjusted\;Mean+\frac{Adjusted\;SD}{Raw\;SD}\times (Raw\;Score-Raw\;Mean)$$

For adjustment of the exam mean only, all the scores were adjusted to the same degree without changing the distribution (SD) of the grades. An example of this type of grade adjustment is when the overall degree of difficulty of the exam (based on the observed item difficulty of the individual questions) is higher than what the instructor intended. Considering the example data presented here (Figure 2), the instructor may have intended an average item difficulty of 0.78 (exam mean of 78%) had all the questions been previously validated with known item difficulties. Therefore, the mean was adjusted to 78% from the raw mean of 73.8% using Equation (1), with the $\frac{Adjusted\;SD}{Raw\;SD}$ ratio in the equation being equal to 1.

Methods that result in a change in both the mean and distribution of exam grades are infrequently justified at the individual course levels. An example of such adjustment could be when there is a course grade reference curve [27], which has been generated from grade data over several years or for several sections of the same course in the same year. In those cases, the grade reference curve is generated by combining grades from across the years or different sections, generating a mean and SD for all the years or sections combined. The grades in a particular year or a particular section may then be adjusted by adjusting both Mean and SD of the exam using Equation (1). As an example, a reference Mean of 78% (instead of the raw mean of 73.8%) and a reference SD of 13% (instead of raw SD of 15.4%) were used here to adjust the grades by this method.

## 3. Results

### 3.1. Grade Adjustment for Individual Questions

Table 1 lists the raw scores along with the adjusted scores for the three approaches of (1) dropping the question, (2) considering the answers of all students to the question as correct, or (3) adding one point (the score for one question) to all students. As demonstrated in Table 1, it is assumed that only 25% (*n* = 5) of students (students 1, 3, 7, 11, and 17) answered one of the questions correctly (i.e., an item difficulty of 0.25). Dropping this question from the exam results in a maximum point of 24 instead of 25. Whereas the question drop increases the exam grades of those who answered it incorrectly, it causes a reduction in the exam grade for those who answered it correctly (Table 1). Further, whereas the negative impact is more drastic for those with the lower exam grades, those with the highest exam grades who missed that question benefit the most from this type of adjustment. When the question is dropped, the mean, max, and min values of the adjusted scores are +2%, +4.0%, and −2.7%, respectively, different from those for the raw data (Table 1). The second approach, to consider all students answered the question correctly, selectively adds the equivalent of one point (4%) to those with the incorrect answer while leaving the grades of those who initially answered the question correctly intact. This approach results in the mean, max, and min values of the adjusted scores being 3%, 4%, and 2% higher than those of the raw scores (Table 1). The third approach, to add an extra point (4%) to all students regardless of how they answered the question, causes the mean, max, and min values of the adjusted scores to increase by 4% (Table 1). The educational justifications for selecting each of the above three approaches are discussed in the Discussion section below in the context of item analysis statistics.

### 3.2. Grade Adjustment for the Entire Exam

The effects of adjustment of the mean (from 73.8% to 78%), without a change in the grade distribution (SD) or in addition to the adjustment of SD (from 15.4% to 13%), on the students’ scores are presented in Table 2. Adjusting the mean of the exam without a change in its distribution results in similar adjustments in scores for all the students. In the example provided here, every score, including min and max, is increased by 4.2% (78–73.8%). However, the distribution of the grades (i.e., the SD) remains the same (15.4%) (Table 2).

In contrast to adjusting the mean only, when both the mean and distribution (SD) of the scores are adjusted to match a grade reference curve, individual students’ scores and min and max values are impacted to different degrees (Table 2). In the example provided, in the presence of a simultaneous increase in mean and decrease in SD, students’ grades at the lower end are increased to a much more significant degree than those of the high performers (Table 2). Although not shown here, a change in SD may change the scores of different students in opposite directions. For example, a reduction in SD without an increase in mean increases the scores of the students at the lower end and decreases the scores of the students at the higher end of the distribution curve.

## 4. Discussion

The value of and emphasis on assigning grades to students’ work is a matter of debate in higher education, leading to the argument that the time and stress associated with grading may distract from more meaningful pedagogical activities and learning [1]. Nevertheless, grades, whether in numerical form, percentages, letters, or pass/fail, are part of the assessment of students’ work in most disciplines in higher education, including health professions. Grading may be carried out using norm-referenced or criteria-referenced methods [3,5]. Norm-referenced grading is based on the relative standing of students in the class and may not reflect students’ true knowledge or ability. “Grading on the curve” [3,4,15,16,17,18,19], which limits the number of letter grades in each category, regardless of the absolute performance of students, is one of the main applications of norm-based grading.

The “grading on the curve” method is mostly associated with large undergraduate courses and is very controversial. On the other hand, criteria-referenced grading, which is more common in graduate and health profession programs, is supposed to reflect student learning or ability [5]. However, even with criteria-referenced grading, there may be situations when the scores for the individual questions or the entire assessment may need adjustment or curving, which is the subject of the current communication. Examples of such situations include when the degree of difficulty of the questions or exams is higher than what was intended by the instructor, when questions are flawed, or when there is a need to normalize the grades for different sections of the same course or across the years.

The data presented in Table 1 indicate that the three potential approaches dealing with the grade adjustment for individual exam questions result in drastically different outcomes for individual students. The question then becomes under which conditions the use of each of these methods is warranted. As shown in Table 3, dropping the question, which negatively impacts the exam grades of the students who answered it correctly, is only advisable when there is a fundamental flaw with the question or its answer(s). This means even those students who received a point for supposedly answering the question correctly were incorrect in their answers and should not have received credit. Alternatively, if the question has multiple correct answers, one could consider all the answers as being correct. Adding an extra (bonus) point to everybody may be the best option if the question difficulty is more than anticipated or desired (e.g., in this case, only 25% of students answered it correctly), but there is nothing fundamentally wrong with the question/answers (Table 3).

Item analysis statistics, including item difficulty and point biserial, may be used as a guide to flag individual questions in the exam for further review [12,13,14] and to decide whether to drop or rescore a question or add a bonus point. The question investigated in Table 1 has an item difficulty of 0.25, which is considered a very difficult question [14]. Additionally, its point biserial, which is a correlation between the correctness of the answer (correct or incorrect) and the students’ scores in the entire exam (Table 1), is equal to −0.315. The negative point biserial indicates a problematic question because more students with poor performance in the entire exam answered this question correctly (Table 1). If further review of the question confirms structural problems with the question and/or the answers, the appropriate action for this question is then to drop the question, as indicated in Table 3. However, if instead of students 1, 3, 7, 11, and 17, students 14, 15, 17, 19, and 20 (Table 1) had answered the question correctly, the item analysis would reveal a difficulty of 0.25 with a very high positive point biserial of 0.578. This scenario indicates a very difficult question that can discriminate between the low and high performers in the exam. In this case, the question may be retained. However, if the very high degree of difficulty was not intentional, the instructor may add a bonus point to all students, including those who answered the question correctly (Table 3). Nevertheless, it has been suggested [14] that questions with item difficulties outside the 0.6–0.9 range and point biserial values <0.15 need reevaluation and potential rescoring.

Besides grade adjustment for individual questions, presented here is a general method (Equation (1)) for adjustment of grades for an entire assessment based on adjustment of the mean and/or SD of the assessment (Table 2). Whereas the adjustment of the mean changes the scores of all the students to the same extent, adjustment of both mean and SD alters the scores and their distribution, thus affecting students with high and low scores differently (Table 2). Educational justifications for this type of adjustment may include adjustments for the higher-than-intended degree of difficulty of an exam because of several new questions with a high degree of difficulty or implementation of a course grade reference curve [27] across the years or different sections of the course (Table 3). Besides these scenarios for the individual courses in the program, grade adjustment for the entire assessment may also be indicated at the program level to rank or compare students for the award of scholarships or entrances to competitive programs, like graduate studies.

In addition to Equation (1), there are other methods available in the literature for the adjustment of the entire exam grades, which mainly deal with the topic of grade “moderation”, used for the General Certificate of Secondary Education in the United Kingdom [20,22] or Higher School Certificates in Australia [21,23]. The primary purpose of these reported grade “moderation” methods is to adjust students’ grades from different schools based on a central assessment administered to all students. These methods may also be applied to adjust grades for the entire assessments for programmatic purposes. For interested educators, an adaptation of these methods to change the mean and SD of the assessments by fixing two (e.g., mean and max) or three (e.g., mean, min, and max) assessment parameters using linear-scale or quadratic polynomial models, respectively, are presented in Appendix A.

Adjustment or curving of the grades for the entire assessment should not be viewed as a simple remedy to increase the grades when a large number of students perform poorly or fail the assessment [1]. In those situations, the instructor should first investigate why students performed poorly by reevaluating the assessment and other pedagogical methods before attempting to curve the exam grade. For example, the instructor should reevaluate the validity of the individual questions and rescore or eliminate those that are flawed, as discussed earlier. Additionally, students may be allowed to revisit questions with high item difficulty and resubmit their answers for some credits as an alternative to grade adjustment. Nevertheless, a serious reevaluation of an assessment with a large number of failures may reveal gaps and deficiencies in the instructor’s pedagogical approaches that cannot be resolved by a simple grade adjustment.

As mentioned earlier, to mimic the grades in most professional programs, the simulated grades used in this study are intentionally skewed toward higher frequency for the higher grades (Figure 1). However, the principles for grade adjustment presented above are equally applicable when lower grades are more frequent or when the grades are normally distributed.

A limitation of this study is that it did not investigate how curving grades affects student performance in subsequent assessments. Future studies should evaluate the impact of adjustment or curving of the grades on student learning.

## 5. Conclusions

In conclusion, different methods for adjustment of assessment scores are presented here for individual questions and/or an entire assessment. Although these methods are relatively easy to implement, instructors should have reasonable educational justification for deciding whether to adjust grades or which method to use. When adjusting the grades is required, instructors should use the item analysis data for adjusting the question grades and mean and/or distribution data across the course sections or years for adjusting the exam grades.

## Figures and Tables

**Table 1:** The effects of three different methods of grade adjustment for addressing a flawed or difficult question on the student scores *^a^*.

Raw Scores				Adjusted Scores								
No Adjustment				Drop One Question			Consider All Correct			Add a Point to All		
Student	Points (25)	%	Correct?	Points (24)	%	Change	Points (25)	%	Change	Points (25)	%	Change
1	9	36	Yes	8	33.3	−2.7	9	36	0	10	40	4
2	13	52	No	13	54.2	2.2	14	56	4	14	56	4
3	14	56	Yes	13	54.2	−1.8	14	56	0	15	60	4
4	15	60	No	15	62.5	2.5	16	64	4	16	64	4
5	15	60	No	15	62.5	2.5	16	64	4	16	64	4
6	16	64	No	16	66.7	2.7	17	68	4	17	68	4
7	17	68	Yes	16	66.7	−1.3	17	68	0	18	72	4
8	18	72	No	18	75.0	3.0	19	76	4	19	76	4
9	19	76	No	19	79.2	3.2	20	80	4	20	80	4
10	19	76	No	19	79.2	3.2	20	80	4	20	80	4
11	20	80	Yes	19	79.2	−0.8	20	80	0	21	84	4
12	20	80	No	20	83.3	3.3	21	84	4	21	84	4
13	20	80	No	20	83.3	3.3	21	84	4	21	84	4
14	20	80	No	20	83.3	3.3	21	84	4	21	84	4
15	21	84	No	21	87.5	3.5	22	88	4	22	88	4
16	21	84	No	21	87.5	3.5	22	88	4	22	88	4
17	22	88	Yes	21	87.5	−0.5	22	88	0	23	92	4
18	22	88	No	22	91.7	3.7	23	92	4	23	92	4
19	24	96	No	24	100	4.0	25	100	4	25	100	4
20	24	96	No	24	100	4.0	25	100	4	25	100	4
**Mean**	**18.5**	**73.8**		**18.2**	**75.8**	**2.0**	**19.2**	**76.8**	**3**	**19.5**	**77.8**	**4**
**Max**	**24.0**	**96.0**		**24.0**	**100**	**4.0**	**25.0**	**100**	**4**	**25.0**	**100**	**4**
**Min**	**9.0**	**36.0**		**8.0**	**33.3**	**−2.7**	**9.0**	**36.0**	**2**	**10.0**	**40.0**	**4**

*^a^* Shaded rows indicate students who answered the question correctly.

**Table 2:** The effects of adjustment of the mean (from 73.8% to 78%) with (SD, 13.0%) and without (15.4%) a change in the grade distribution (SD) *^a^*.

Raw Scores		Adjusted Scores			
No Adjustment		Adjusting Mean Alone		Adjusting Mean and SD	
Student	Score, %	Score, %	Change, %	Score, %	Change, %
1	36	40.2	4.2	46.1	10.1
2	52	56.2	4.2	59.6	7.6
3	56	60.2	4.2	63.0	7.0
4	60	64.2	4.2	66.3	6.3
5	60	64.2	4.2	66.3	6.3
6	64	68.2	4.2	69.7	5.7
7	68	72.2	4.2	73.1	5.1
8	72	76.2	4.2	76.5	4.5
9	76	80.2	4.2	79.9	3.9
10	76	80.2	4.2	79.9	3.9
11	80	84.2	4.2	83.2	3.2
12	80	84.2	4.2	83.2	3.2
13	80	84.2	4.2	83.2	3.2
14	80	84.2	4.2	83.2	3.2
15	84	88.2	4.2	86.6	2.6
16	84	88.2	4.2	86.6	2.6
17	88	92.2	4.2	90.0	2.0
18	88	92.2	4.2	90.0	2.0
19	96	100.2	4.2	96.8	0.8
20	96	100.2	4.2	96.8	0.8
**Mean**	**73.8** *^b^*	**78.0** *^b^*	**4.2**	**78.0** *^c^*	**4.2**
**Max**	**96.0**	**100.2**	**4.2**	**96.8**	**10.1**
**Min**	**36.0**	**40.2**	**4.2**	**46.1**	**0.8**

*^a^* Using Equation (1) in the text. *^b^* SD of 15.4%. *^c^* SD of 13.0%.

**Table 3:** Summary of different grade adjustment methods for individual questions or the entire assessment.

**Grade Adjustment for Individual Questions**	
**Method**	**Use**
1. Drop the question	When the question and/or answers are fundamentally flawed.
2. Consider all answers correct	When the other answers are also correct.
3. Add an extra point to all	When there is nothing wrong with the question or answers, but the item difficulty is more than anticipated or desired.
**Grade Adjustment for the Entire Exam**	
**Method**	**Use**
1. Adjusting the mean	When it is desired to change the grades of all students to the same extent, such as when the overall degree of difficulty of the exam is higher than intended.
2. Adjusting mean and distribution	When it is desired to use a grade reference curve to have consistency in grading across the years or several sections of the course.

**Figure A1:**
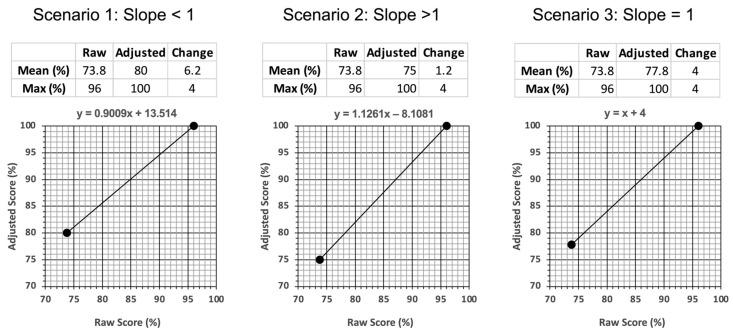
Examples of adjustment of mean and max scores for three scenarios, resulting in slopes of < 1 (**left panel**), >1 (**middle panel**), and 1 (**right panel**) when adjusted scores are plotted against the raw scores. The equations on top of the graphs are generated by the trendline function of the Excel program.

**Figure A2:**
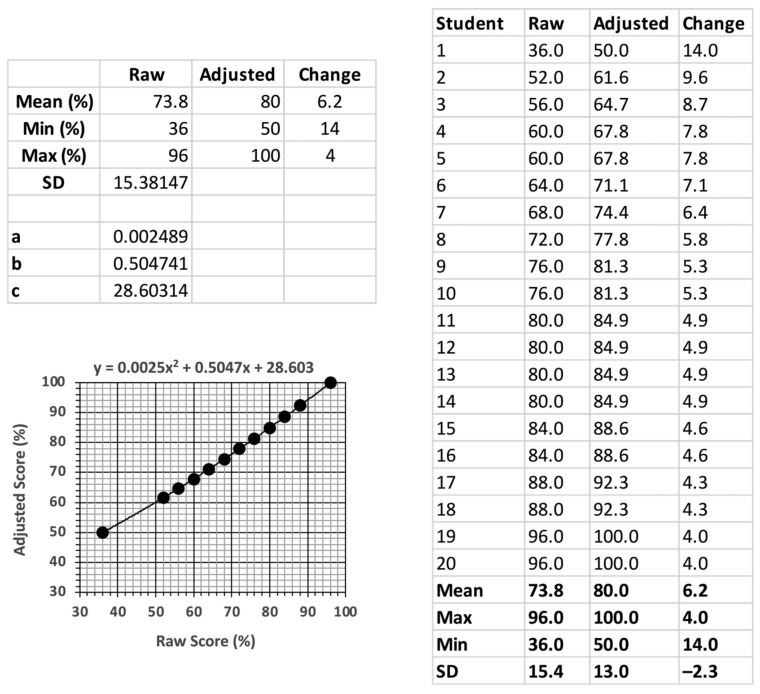
An example of the effects of adjustment of mean, min, and max scores on the student scores. The values of *a*, *b*, and *c* are estimated from Equations (A3)–(A5), respectively, in Appendix A.

## Data Availability

Data are contained within the article or Supplementary Materials.

## Supplementary Materials

The following supporting information can be downloaded at https://www.mdpi.com/article/10.3390/pharmacy13010004/s1: An Excel^®^ file containing the grade adjustment methods for fixing mean and/or SD and fixing two (e.g., mean and max) or three (e.g., mean, min, and max) exam parameters.

## Appendix A

### Appendix A.1. Grade Adjustment by Fixing Two Exam Parameters (e.g., Mean and Max)

This method is based on a simple, linear-scale model to adjust the exam grades for all students. The model is explained in the following steps:

- Select the desired mean (adjusted mean) and max (adjusted max) scores.
- Plot the desired (adjusted) values against their corresponding raw values in an Excel^®^ spreadsheet to obtain the slope and intercept of the trendline defining the relationship between the adjusted (y-axis) and raw (x-axis) scores, as shown in the following equation:
  (A1) Adjusted Score=Intercept+Slope×Raw Score
- Use the slope and intercept in the above equation to calculate an adjusted score for each raw score.

Theoretically, the slope in the above equation could be <1 (Scenario 1), >1 (Scenario 2), or equal to 1 (Scenario 3), depending on the selected values of the adjusted scores relative to their corresponding raw scores, as shown in Figure A1.

In these scenarios, the mean and max scores are set to values desired by the instructor. For example, in Scenario 1, mean is increased by 6.2% (from raw mean of 73.8% to adjusted mean of 80%) and max is increased by 4% (from raw max of 96% to adjusted max of 100%), and the following linear equation describes the relationship between the two data sets:

Adjusted Score=13.514+0.9009×Raw Score

The above equation is then used to calculate the adjusted scores for each student in this scenario (Scenario 1). The raw scores and their adjusted scores for all three scenarios are listed in Table A1. As demonstrated in the table, with a slope of <1 (Scenario 1), the students with lower scores receive higher increases in their scores. However, when the slope is larger than 1 (Scenario 2), the opposite is true, which means the students with the higher raw scores receive the larger positive adjustments. Additionally, for Scenario 2, depending on the magnitude of the slope and the range of raw scores, the scores of the students with the lower raw scores may be adjusted downward (Table A1). For Scenario 3, when the slope is equal to 1, the magnitude of the adjustment is the same for all the students (Table A1), regardless of the value of their raw scores, a scenario that is identical to fixing the mean only or adding points to all students.

**Table A1:** The effects of linear adjustment of mean and max scores on the student scores when the slope of the adjusted versus raw score line is <1, >1, or 1 *^a^*.

Raw Scores		Adjusted Scores					
No Adjustment		Scenario 1: Slope < 1		Scenario 2: Slope > 1		Scenario 3: Slope = 1	
Student	Score, %	Score, %	Change, %	Score, %	Change, %	Score, %	Change, %
1	36	45.9	9.9	32.4	−3.6	40	4
2	52	60.4	8.4	50.5	−1.5	56	4
3	56	64.0	8.0	55.0	−1.0	60	4
4	60	67.6	7.6	59.5	−0.5	64	4
5	60	67.6	7.6	59.5	−0.5	64	4
6	64	71.2	7.2	64.0	0.0	68	4
7	68	74.8	6.8	68.5	0.5	72	4
8	72	78.4	6.4	73.0	1.0	76	4
9	76	82.0	6.0	77.5	1.5	80	4
10	76	82.0	6.0	77.5	1.5	80	4
11	80	85.6	5.6	82.0	2.0	84	4
12	80	85.6	5.6	82.0	2.0	84	4
13	80	85.6	5.6	82.0	2.0	84	4
14	80	85.6	5.6	82.0	2.0	84	4
15	84	89.2	5.2	86.5	2.5	88	4
16	84	89.2	5.2	86.5	2.5	88	4
17	88	92.8	4.8	91.0	3.0	92	4
18	88	92.8	4.8	91.0	3.0	92	4
19	96	100.0	4.0	100.0	4.0	100	4
20	96	100.0	4.0	100.0	4.0	100	4
**Mean**	**73.8**	**80.0**	**6.2**	**75.0**	**1.2**	**77.8**	**4**
**Max**	**96.0**	**100.0**	**4.0**	**100**	**4.0**	**100**	**4**
**Min**	**36.0**	**45.9**	**9.9**	**32.4**	**−3.6**	**40.0**	**4**

^*a*^ See Figure A1 for the definition of scenarios.

Although mean and max scores were adjusted in the example here, the two scores chosen for the adjustments could be any scores from the raw scores. So, in addition to the above example, one may adjust mean and min or min and max.

### Appendix A.2. Grade Adjustment by Fixing Three Exam Parameters (e.g., Mean, Min, and Max)

Adjusting the three parameters of mean, min, and max is one of the more complex procedures for grade adjustments, which creates a nonlinear polynomial relationship between the adjusted and raw scores, as described below:

(A2) $$Adjusted\;Score=a\cdot {Raw\;Score}^{2}+b\cdot Raw\;Score+c$$

The coefficients *a* and *b* and constant *c* in the above equation may be calculated from the raw and adjusted values of mean, min, and max and the SD of the raw scores, as shown below [23]:

(A3) $$a=\frac{Adjusted\;Max\cdot \left(Raw\;Min-Raw\;Mean\right)-Adjusted\;Min\cdot \left(Raw\;Max-Raw\;Mean\right)+Adjusted\;Mean\cdot (Raw\;Max-Raw\;Min)}{\left(Raw\;Max-Raw\;Min\right)\cdot [\mathrm{Raw}{SD}^{2}+\left(Raw\;Max-Raw\;Mean\right)\left(Raw\;Min-Raw\;Mean\right)]}$$

(A4) $$b=\frac{\left(Adjusted\;Min-Adjusted\;Mean)-a\cdot ({Raw\;Min}^{2}-{Raw\;Mean}^{2}-{Raw\;SD}^{2}\right)}{\left(Raw\;Min-Raw\;Mean\right)}$$

(A5) c=Adjusted Min−Raw Min·(a×Raw Min+b)

Although *a*, *b*, and *c* values may be estimated using calculators, a spreadsheet with embedded formulas can easily automate these calculations (see Supplementary File).

Figure A2 shows an example with adjusted mean, min, and max values of 80%, 50%, and 100%, compared with the raw values of 73.8%, 36%, and 96%, respectively. The calculated values of coefficients *a* and *b* and constant *c* are 0.002489, 0.504741, and 28.60314, respectively, resulting in the following relationship that was used for the calculation of the adjusted scores for every student:

$$Adjusted\;Score=0.002489\cdot {Raw\;Score}^{2}+0.504741\cdot Raw\;Score+28.60314$$

As shown in Figure A2, the relationship between the adjusted score and raw score has an upward concave shape, showing higher slopes at higher scores. This is because the value of coefficient *a* is positive. Similar to Scenario 1 for the linear adjustment of two parameters, the upward concave relationship with the adjustment of three parameters results in higher adjustments for students with lower scores (Figure A2). The quadratic polynomial relationship would collapse to the linear adjustment described above under conditions that result in the value of zero for coefficient *a*. For example, if in the above example, the adjusted min is set to 45.9% instead of 50%, the above quadratic polynomial relationship collapses to the following linear relationship that is almost identical to Scenario 1 in Figure A1 and Table A1:

Adjusted Score=13.424+0.9021×Raw Score
